# Impact of the time interval between biopsy and radical prostatectomy on functional outcomes

**DOI:** 10.1007/s00345-024-05324-3

**Published:** 2024-11-16

**Authors:** Rosannis Brown, Burkhard Beyer, Sophie Knipper, Gisa Mehring, Lars Budäus, Pierre Tennstedt, Markus Graefen, Randi M. Pose

**Affiliations:** 1 Martini-Klinik Prostate Cancer Centre, University Hospital Hamburg-Eppendorf, Hamburg, Germany; 2 Department of Urology, University Hospital Hamburg-Eppendorf, Martinistraße 52, 20246 Hamburg, Germany; 3 Clinic Wildetal, Urological Competence Centre for Rehabilitation, Kliniken Hartenstein, Bad Wildungen, Germany; 4 Department of Urology, Vivantes Klinikum Am Urban, Berlin, Germany

**Keywords:** Prostate cancer, Radical prostatectomy, Continence, Potency, Functional outcomes

## Abstract

**Purpose:**

The aim of our study was to investigate the impact of the time interval between prostate biopsy and radical prostatectomy (RP) on postoperative urinary continence (UC)/erectile function (EF). From a clinical point of view, an interval of several weeks seems to facilitate surgical preparation.

**Materials and methods:**

We retrospectively analyzed patients who underwent RP for localized prostate cancer (PCa) in a tertiary care center between 2011 and 2020. We evaluated the influence of the following variables on UC and EF 1 year after RP: time from biopsy to RP, age, BMI, pathological T-stage, EF and intraoperative nerve sparing (unilateral vs. bilateral). For this purpose, we performed linear regression analyses as well as manual grouping and cluster analyses to identify possible temporal cutoff ranges. The EPIC-26 and the IIEF questionnaires were used for the assessment of UC and EF.

**Results:**

We identified 6202 consecutive patients who underwent RP. Neither manual grouping nor cluster analyses showed a significant difference in continence or potency after RP. According to linear regression models, only age was an independent predictor of incontinence (95%-CI 0.006–0.01), and EF before RP (95%-CI 0.22–0.26), age (95%-CI – 0.68 to – 0.5), BMI (95%-CI – 0.66 to – 0.29) and bilateral NS (95%-CI 5.5–2.1) had significant impacts on postoperative EF (all p < 0.001).

**Conclusion:**

In the selected patient population, the time interval between prostate biopsy and RP did not seem to have an effect on postoperative functional outcomes (UC and EF).

**Supplementary Information:**

The online version contains supplementary material available at 10.1007/s00345-024-05324-3.

## Introduction

Radical prostatectomy (RP) is a curative treatment option for localized prostate cancer (PCa) [1]. The primary therapeutic goal, optimal tumor control, is followed by optimal functional outcomes, especially preservation of urinary continence (UC) and potency (erectile function [EF]). In recent decades, different factors have been found to reduce surgery-associated morbidity. These factors include noninfluencing factors such as patient age, pathologic tumor stage, prostate size, preoperative UC, and knowledge of important anatomic structures [2–4]. The influence of different surgical approaches (i.e., open vs. robotic surgery) on functional outcomes is still controversial [5–7]. However, technical factors such as nerve sparing (NS) and complete sphincter preservation have led to significant postoperative improvements [3, 4]. Moreover, preoperative invasive manipulations of the prostate, such as transurethral resection of the prostate (TURP), as well as the time interval between manipulations and RP, seem to have a negative influence on functional outcome after prostatectomy [8, 9].

Prostate biopsy is considered the gold standard for confirming the histological diagnosis and planning treatment of PCa [1]. However, biopsies are associated with some risk of postinterventional infections, temporary erectile dysfunction and micturition disorders [10–12]. Therefore, in clinical practice, adhering to certain recovery times between biopsy and RP is sometimes advised to avoid negatively impacting surgical outcomes through inflammatory tissue changes. However, whether UC and EF after RP are affected solely by the invasiveness of the biopsy itself is difficult to investigate due to the indispensability of a diagnostic biopsy. The literature on this topic is sparse, based on small or historical cohorts and limited by the unstandardized assessment of functional outcomes. Therefore, we hypothesized the following:

The time interval between specimen collection and prostatectomy may affect functional outcome. We aimed to perform a comprehensive contemporary analysis in this study to test our hypothesis in a large cohort.

## Materials and methods

### Study cohort/patient population

We retrospectively analyzed 23,074 consecutive patients who underwent open retropubic (ORP) or robotic-assisted RP (RARP) for localized PCa at a high-volume tertiary care center between 2011 and 2020. All RPs were performed by high-volume surgeons for both surgical approaches. All included patients had undergone exact one single biopsy before RP (n = 16,445) and reported preoperative urinary continence (no need for a pad, n = 16,363); we also included only patients who underwent unilateral or bilateral nerve-sparing surgery (n = 15,237) as well as patients who had received neither (neo)adjuvant androgen deprivation therapy (n = 13,564) nor (salvage) radiotherapy (n = 11,331), as these can have negative effects on continence and erectile function [13]. The basic requirement for inclusion in the study was completion of standardized questionnaires before and after RP via patient-reported outcome measures (PROMs, n = 6455). We also excluded patients whose biopsy time interval was more than 1000 days. This resulted in a final study cohort of 6,202 patients. The detailed case selection process is shown in the cohort flow chart (Fig. 1 Supplements).

The RPs were generated according to standardized procedures and using the full-functional-length-urethra (FFLU) surgical technique mentioned above and the neuroSAFE technique/neurovascular structure-adjacent frozen-section examination (NeuroSAFE) [4, 14, 15].

All patients provided informed consent for the procedure, data collection, and data analysis. This approach permits the collection of deidentified patient data at baseline and follow-up, which were entered into a secure, password-protected database for subsequent analysis. This retrospective analysis was approved by the institutional review boards. All men signed an informed consent form on data collection. All the data were prospectively stored in an institutional database (FileMaker Pro 11; Claris, Inc.).

### Definition of outcome variables

The analyses focused on postoperative functional outcomes (UC and EF). PROM data were collected preoperatively, one month post-RP, six months post-RP, and twelve months post-RP. UC was measured by individual daily pad use, which was assessed using the Expanded Prostate Cancer Index Composite (EPIC)-26 questionnaire [16]. Here, the use of no or one safety pad/24 h was defined as continent, and the regular use of one or more pads/24 h was defined as incontinent.

Potency was defined according to IIEF question 2 (score of 4 or 5) and EPIC-26 question 9 (score of 100%). EF was also defined as a score of ≥ 75 on the EPIC 26 (The Expanded Prostate Cancer Index Composite, Sexual Assessment) sexual item.

In addition, the date of initial PCa diagnosis, date of biopsy, initial PSA level, surgical procedure, T stage, lymph node status, and nerve preservation (unilateral vs. bilateral) were recorded for each patient.

### Statistical analyses

We analyzed the influence of the following predictors on functional outcomes: patient age (continuously coded and categorized), BMI (continuously coded and categorized), pathological T stage (pT3a and pT3b versus (vs.) pT2), time from biopsy to RP (continuously coded and categorized), and intraoperative nerve sparing (unilateral vs. bilateral).

Descriptive statistics included frequencies and proportions for categorical variables (pathological T-stage, N-stage, nerve sparing). Means, medians, and ranges are reported for continuously coded variables (age, BMI, prostate volume, PSA concentration, number of cores, days between biopsy and RP). The chi-square test was used to test for the statistical significance of differences between proportions. The t test and Mann‒Whitney U test were used to evaluate the statistical significance of the means and median differences, respectively.

To examine correlations, days from biopsy to RP were grouped by clinical significance, and additional linear regression analyses were performed for continence (24-h pad use) and potency (changes in IIEF or the EPIC-26 score). Hierarchical cluster analysis was performed to further search for correlations. Here, the function *daisy* from the R package *cluster* was used to calculate pairwise dissimilarities between the observations. Moreover, the function *hclust* from the R package *stats* was used for hierarchical clustering.

To visualize the distribution of postoperative potency and continence among patients according to the time interval between biopsy and surgery, we generated a density plot. The table shows values that are calculated using a kernel density estimate (KDE). This kernel is the standard Gaussian kernel for the normal distribution. The values on the y-axis can reach a maximum of 1 and are dependent on the number of cases per data point on the x-axis.

All the statistical tests were two-sided, and the significance level was set at p < 0.05. Analyses were performed using the R software environment for statistical computing and graphics (version 4.1.2) and RStudio (Posit Software, PBC) [17].

## Results

In total, we analyzed 6,202 patients. The basic patient characteristics are shown in Table 1.

**Table 1:** Basic patient characteristics (n = 6202) who underwent radical prostatectomy with nerve sparing from 2011 to 2020 in our tertiary care center after only one prior biopsy

Patients	n	6202
Age at RP (years)	Median (IQR)	63 (58–68)
PSA (ng/ml)	Median (IQR)	6.7 (5–9.4)
BMI (kg/m^2^)	Median (IQR)	26.3 (24.4–28.4)
Days Bx—RP	Median (IQR)	77 (55–107)
Prostate volume (cm.^3^)	MEDIAN (IQR)	36 (28–50)
Number biopsy cores	MEDIAN (IQR)	10 (8–12)
Number positive cores	MEDIAN (IQR)	3 (2–5)
Surgical approach	n (%)	
ORP		3382 (54.5)
RARP		2820 (45.5)
pT stage	n (%)	
pT2		4669 (75.4)
pT3a		1241 (20)
≥ pT3b		285 (4.6)
pN status	n (%)	
NX		928 (15)
N0		5067 (81.8)
N1		199 (3.2)
Nerve sparing	n (%)	
Bilateral		5061 (81.6)
Unilateral		1141 (18.4)
Surgical margin	n (%)	
R0		5623 (90.8)
R1		573 (9.2)

*BMI* body mass index, *BX* biopsy, *n* number, *ORP* open radical prostatectomy, *R0* negative surgical margin, *R1* positive surgical margin, *RARP* robot-assisted radical prostatectomy, *RP* radical prostatectomy

The median patient age was 63 years (interquartile range (IQR) 58–68 years), the median initial PSA level was 6.7 ng/ml (IQR 5–9.4 ng/ml), the median prostate volume was 36 cm^3^ (IQR 28–50 cm^3^) and the median body mass index (BMI) was 26.3 kg/m^2^ (IQR 24.4–28.4 kg/m^2^). A median number of 10 cores (IQR 8–12 cores) were taken, of which a median of 3 (IQR 2–5 cores) were positive. The median time from biopsy to RP was 77 days (IQR 55–107 days). ORP was performed in 3382 (54.5%) patients, and RARP was performed in 2820 (45.5%) patients. Bilateral nerve-sparing RP was performed in 5061 (81.6%) patients. A histopathological workup of the prostatectomy specimens revealed negative surgical margins (R0 resection) in 5623 (90.8%) patients and organ-confined tumors (pT2) in 4669 (75.4%) patients. In 5067 (81.8%) patients the lymph node status was negative and in 928 (15%) patients lymphadenectomy was not necessary due to the tumor characteristics (Table 1). One year after RP, 88.3% of patients reported complete UC (no pad or use of one safety pad per 24 h), and the rate of postoperative erectile dysfunction (ED) was 81.8% (defined as an EPIC-26 score < 75) (Table 3, Supplements) [18].

We graphically examined the relationship between pad consumption and the number of days between biopsy and surgery using a density-plot, which visually revealed a slight increase in pad consumption during early surgeries after biopsy (Fig. 2, Supplements). Table 4 (Supplements) shows the median and IQR days since biopsy to RP stratified after pad use and potency one year after RP. However, no significant difference was found in the calculated analyses.

According to clinical experience, manual groupings were created for the temporal intervals between biopsy and RP (RP ≤ 28 days, RP 29–99 days and RP ≥ 100 days after biopsy; Table 5, Supplements). Here, no significant differences were observed with respect to postoperative UC or EF at one year. Even after adjusting for grouping (RP up to 30 days, RP 31–60 days, RP 61–100 days, and RP > 100 days), no significant differences were found.

Moreover, cluster analyses revealed no statistically significant delineable temporal intervals (between biopsy and RP) that had an impact on patients’ postoperative functional outcomes (UC and EF).

Table 2 shows the results of multivariable linear regression analysis for pad consumption per 24 h and postoperative potency. Of the variables listed, only patient age had a significant effect on continence (95%-CI: 0.0056—0.0095, p < 0.001). Independent predictors of postoperative potency were a high score on the IIEF and EPIC-26 sexual items before RP (95% CI: 0.2188—0.2583, p < 0.001), patient age at the time of RP (95% CI: -0.6799—-0.4964, p < 0.001), BMI (95% CI: -0.6555—-0.2853, p < 0.001) and bilateral nerve sparing (95% CI: 5.5429—2.0557, p < 0.001). The number of days between biopsy and RP had no significant effect on functional outcome.

**Table 2:** Multivariable linear regression analysis for the prediction of urinary incontinence (usage of ≥ 1 pad per day) 1 month, 6 months and 1 year after radical prostatectomy and for the prediction of erectile function (EPIC-26 score) 6 months and 1 year after RP

Variable	1 month			6 months			1 year		
	Estimates	95% CI	p value	Estimates	95% CI	p value	Estimates	95% CI	p value
*Incontinence*									
Days BX until RP	0	– 0.0004 to 0.0004	0.9	0	– 0.0003 to 0.0003	0.9	0	– 0.0002 to 0.0002	0.9
Age	0.02	0.018–0.027	< 0.001	0.01	0.007–0.014	< 0.001	0.008	0.006–0.01	< 0.001
BMI	0.01	0.003–0.021	0.009	0.007	– 0.001 to 0.014	0.07	0.003	– 0.001 to 0.007	0.1
pT3a	0.05	– 0.036 to 0.126	0.3	– 0.04	– 0.1 to 0.027	0.3	– 0.03	– 0.065 to 0.008	0.1
≥ pT3b	– 0.07	– 0.222 to 0.078	0.4	– 0.03	– 0.137 to 0.083	0.6	– 0.02	– 0.088 to 0.048	0.6
NS unilateral	0.17	0.083–0.25	< 0.001	0.05	– 0.013 to 0.12	0.1	0.03	– 0.012 to 0.06	0.2
*Erectile function*									
Days BX until RP	–	–	–	0.001	– 0.006 to 0.007	0.9	0.001	– 0.007 to 0.01	0.7
EPIC Sex Score before RP	–	–	–	0.2	0.134–0.167	< 0.001	0.2	0.22–0.26	< 0.001
Age	–	–	–	– 0.4	– 0.44 to – 0.29	< 0.001	– 0.6	– 0.68 to – 0.5	< 0.001
BMI	–	–	–	– 0.2	– 0.32 to – 0.014	0.03	– 0.5	– 0.66 to – 0.29	< 0.001
pT3a	–	–	–	– 1.7	– 3.1 to – 0.34	0.01	– 1.5	– 3.2 to 0.19	0.08
≥ pT3b	–	–	–	– 3.2	– 5.8 to – 0.67	0.01	– 3.2	– 6.33 to – 0.09	0.04
NS unilateral	–	–	–	– 2.1	– 3.5 to – 0.66	0.004	– 3.8	– 5.54 to – 2.1	< 0.001

*BX* biopsy, *BMI* body mass index, *NS* nerve sparing, *RP* radical prostatectomy

## Discussion

Currently, a prostate biopsy is required to confirm the diagnosis of PCa [1]. From clinical experience, a short time interval between biopsy and RP seems to be associated with an increased incidence of adhesions of the capsule with the prostate [2, 19]. In the daily routine of our tertiary center, intraoperative nerve sparing seems to be technically more difficult in the presence of adhesions. Previous studies have shown that extrinsic factors such as TURP before RP or adjuvant/salvage radiotherapy of the prostate have a significant negative impact on postoperative continence [8, 13]. However, the literature regarding the best time interval to perform RP after biopsy is scarce.

Our study on the temporal interval between biopsy and RP contains some remarkable findings. First, it was not possible to find a cutoff time that would set a significant time limit (time between biopsy and RP) to achieve better functional outcomes (neither continence nor potency) one year after surgery. Thus, according to our analyses, the time interval between biopsy and RP does not seem to have a significant impact on the postoperative functional outcome one year after RP.

Because we expected differences in functional outcome depending on the time interval between biopsy and RP based on clinical experience, the results of manual clustering (days biopsy to RP) were modified according to clinical empiricism (≤ 28 days, 29–99 days and ≥ 100 days as well as ≤ 30 days, 31–60 days, 61–100 days and ≥ 100 days). Due to the nonsignificant findings, we also performed multiple cluster analyses. However, no significant differences were found in the cluster analyses or in the manual groupings.

Here, the 6,202 included patients ultimately represented a cohort with favorable tumor parameters due to the exclusion criteria (no neoadjuvant therapy, no adjuvant or salvage therapy [radiotherapy or ADT]), i.e., with a very low proportion of tumors > pT3a (4.6%), with positive lymph node status (N1 status: 3.2%), or positive resection margins (R1: 9.2%). All of the analyzed patients were preoperatively continent, and at least partial nerve sparing was performed. The influence of biopsy-related complications on surgical outcomes could not be assessed in this study, as only the minority of patients underwent in-house biopsies, and precise information on complication rates was lacking. All in-house biopsies were performed in accordance with European guidelines, using antibiotic prophylaxis and rectal cleansing with povidone-iodine. However, no reliable data were available regarding the procedures used for biopsies conducted externally. Therefore, no conclusions could be drawn regarding the biopsy approach (transrectal vs. transperineal).

Compared to those in the recently published comparator cohort, the patients in our cohort were of comparable age and nutritional status (BMI) at the time of RP; therefore, the aforementioned potential positive selection of our cohort was mainly due to tumor and treatment parameters [6]. This selection was made deliberately to minimize confounding factors for functional outcome (T4 tumors, radiotherapy) and thus not negatively affect the results.

In 2019, Westerman et al. reported 7,350 men after RP (1994–2012) with clinically localized PCa [20]. According to their findings, men who underwent RP ≤ 3 weeks after biopsy had significantly more incontinence (8.7% vs. 5.8%; p < 0.001) and worse potency (31.7% vs. 68.2%; p < 0.001) than those who underwent RP > 12 weeks after biopsy [20]. Unfortunately, it is unclear how many patients the functional outcomes of their study were based on and how they assessed these outcomes. Presumably, no standardized PROM was used. Furthermore, in this historical cohort, the reported continence rates before the introduction of the FFLU technique, as well as the potency rates with unclear nerve sparing and a T4 proportion of just under 10%, appear relatively high [3, 15]. Even with high-volume surgeons, "only" 50% of patients are expected to have an IIEF-5 score ≥ 18 (higher in RARP patients and lower in ORP patients) one year after surgery [6].

In line with our findings, Lee et al. described no impact of the time interval between biopsy and RP on functional outcomes in their 2005 published cohort of 169 patients [21]. Due to the weakness and inconsistency of the collected data, Li et al. did not further analyze postoperative continence or potency in their meta-analysis of the time interval [22].

Our study is not devoid of limitations. First, this study is limited by the retrospective approach of the analyses. Furthermore, the waiting time between Bx and RP was variable. For example, the time interval between biopsy and RP depended on the age and preexisting disease profile of the patients, which sometimes required risk assessment by cardiological diagnostics and, if necessary, interventions before RP. In addition, it cannot be precisely determined what proportion of the patients were on active surveillance without course biopsy (off-label) before RP, and the decision was made to undergo surgery after a prolonged time interval.

Moreover, our analyses are largely based on PROM data. Because not all patients responded to our questionnaires pre- and postoperatively, there was a relevant failure rate whose functional outcomes (postoperative EF and UC) could not be determined (resulting in “only” 6,202 patients). Further positive selection by higher response rates of continent and potent (and thus potentially satisfied) patients cannot be excluded.

Additionally, all patients underwent PR at a tertiary care center for high-volume surgeons. It is well known that functional outcome after PR may be influenced by the experience and operative skills of the surgeon [6]. Therefore, the results of our study may not be generalizable to all surgically treated PCa patients.

## Conclusion

In this selected preoperatively continent patient population with relatively favorable tumor parameters, the time interval between prostate biopsy and RP did not seem to have an effect on functional outcomes (UC and EF) after surgery. Therefore, surgical therapy seems to be feasible even in the short term after biopsy without deterioration of functional outcomes.

## Supplementary Information

Below is the link to the electronic supplementary material.

Supplementary file1 (DOCX 288 KB)

## Data Availability

The data that support the findings of this study are available from the corresponding author, [R.B.], upon reasonable request.
