# Supplementary material for: Impact of the time interval between biopsy and radical prostatectomy on functional outcomes
Source: World J Urol. 2024 Nov 16;42(1):640. doi: 10.1007/s00345-024-05324-3 (PMC11569003; doi:10.1007/s00345-024-05324-3)
Supplement: Supplementary file 1 — Supplementary file1 (DOCX 288 KB) [file 345_2024_5324_MOESM1_ESM.docx]

**Supplements**

**“Impact of the time interval between biopsy and radical prostatectomy on functional outcomes”**

World Journal of Urology

Corresponding author: Rosannis Brown, MD

Department of Urology

University Hospital Hamburg-Eppendorf

Martinistraße 52, 20246 Hamburg, Germany

rosebrow@gmx.de

Table 3: Pad usage per day one month, six months and one year post-RP and potency (determined by the EPIC-26 score) before RP, six months^*^, and one year after RP^*^ _*Postoperative potency for patients with EPIC-26 ≥75 before RP_

| Variable |  | Pre RP | 1 month | 6 months | 1 year |
| --- | --- | --- | --- | --- | --- |
|  |  |  |  |  |  |
| 0/1 safety-pad | n (%) |  | 1136 (22.2) | 2747 (82.8) | 5476 (88.3) |
| 1 pad | n (%) |  | 1927 (37.7) | 289 (8.7) | 430 (6.9) |
| 2 pads | n (%) |  | 768 (15) | 198 (6) | 204 (3.3) |
| ≥ 3 pads | n (%) |  | 1282 (25.1) | 83 (2.5) | 92 (1.5) |
|  |  |  |  |  |  |
| potent* | n (%) | 3853 (71.1) | - | 265 (17.9) | 584 (33) |
| impotent* | n (%) | 1567 (28.9) | - | 1216 (82.1) | 1187 (67) |
|  |  |  |  |  |  |
| SX score | median (IQR) | 43 (0 – 83.3) | - | 18 (0 – 48.7) | 34.7 (0 – 65.3) |
| SX Score ≥ 75 | % | 33.6 | - | 9.8 | 18.2 |

n = number; RP = radical prostatectomy; SX = EPIC-26 sexuality score

* Potency defined according to IIEF question 2 and EPIC-26 question 9

Table 4: Days since biopsy to radical prostatectomy (RP) stratified by pad use and potency one year after RP.

| Variable |  | Median | Q 25% | Q 75% |
| --- | --- | --- | --- | --- |
|  |  |  |  |  |
| 0/1 safety-pad | days | 190 | 98 | 339 |
| 1 pad | days | 98 | 58 | 145 |
| 2 pads | days | 84.5 | 55.2 | 125.8 |
| ≥ 3 pads | days | 81 | 53.5 | 130.5 |
|  |  |  |  |  |
| potent | days | 82 | 56.5 | 111 |
| impotent | days | 84 | 62 | 117 |

Table 5: For example, the manual grouping of days since biopsy up to radical prostatectomy according to clinical significance.

| Variable |  | Grouping of days since biopsy until radical prostatectomy | | |
| --- | --- | --- | --- | --- |
|  |  | ≤ 28d | 29 -– 99d | > 100d |
| BX – RP (d) | Median (IQR) | 23 (19 – 27) | 66 (51 – 81) | 131 (112 – 164) |
| Pads after 1 year |  |  |  |  |
| 0/1 safety-pad | n (%) | 239 (90.2) | 3602 (88.2) | 1635 (88.3) |
| 1 pad | n (%) | 14 (5.3) | 286 (7) | 130 (7) |
| 2 pads | n (%) | 8 (3) | 146 (3.6) | 50 (2.7) |
| ≥ 3 pads | n (%) | 4 (1.5) | 51 (1.2) | 37 (2) |
| Potency after 1 year |  |  |  |  |
| impotent | n (%) | 34 (60.7) | 730 (66.5) | 423 (68.6) |
| potent | n (%) | 22 (39.3) | 368 (33.5) | 194 (31.4) |

BX = biopsy; d = days; n = number; RP = radical prostatectomy


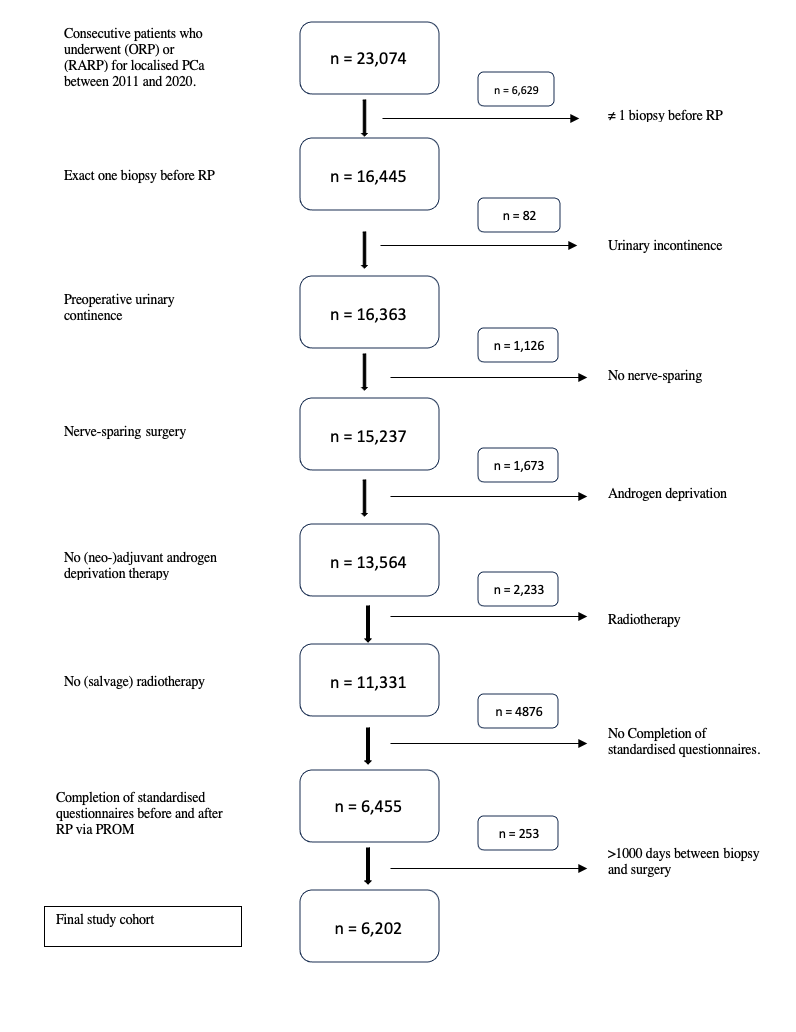


Figure 1: Cohort flow chart


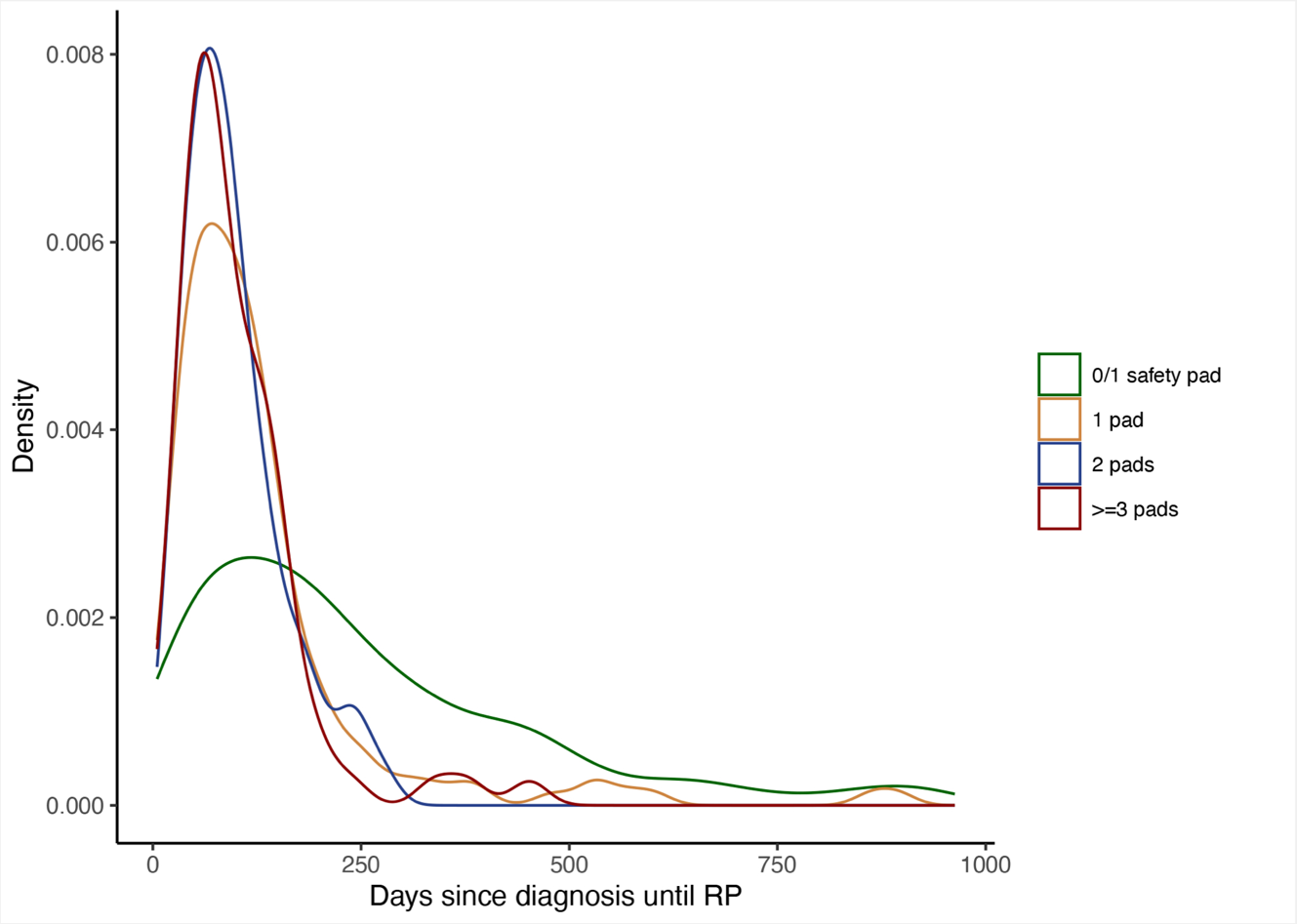


Figure 2: Proportional patient distributions according to pad use one year after surgery and days between biopsy and radical prostatectomy (RP). RP was performed between 2011 and 2020 after only one prior biopsy. The black arrow indicates the peak of patients with a 0/1 safety pad.
